# Stealth Liposomes (PEGylated) Containing an Anticancer Drug Camptothecin: In Vitro Characterization and In Vivo Pharmacokinetic and Tissue Distribution Study

**DOI:** 10.3390/molecules27031086

**Published:** 2022-02-06

**Authors:** Durgaramani Sivadasan, Muhammad H. Sultan, Osama Ali Madkhali, Shahd Hassan Alsabei, Asia Abdullah Alessa

**Affiliations:** Department of Pharmaceutics, College of Pharmacy, Jazan University, Jazan P.O. Box 114, Saudi Arabia; mhsultan@jazanu.edu.sa (M.H.S.); omadkhali@jazanu.edu.sa (O.A.M.); shahdhassan1416@gmail.com (S.H.A.); asia5779@gmail.com (A.A.A.)

**Keywords:** camptothecin, lipid film hydration, polyethylene glycol, encapsulation efficiency, colorectal cancer, pharmacokinetic study, stealth liposomes

## Abstract

Numerous attempts to overcome the poor water solubility of cam ptothecin (CPT) by various nano drug delivery systems are described in various sources in the literature. However, the results of these approaches may be hampered by the incomplete separation of free CPT from the formulations, and this issue has not been investigated in detail. This study aimed to promote the solubility and continuous delivery of CPT by developing long-lasting liposomes using various weights (M.W. 2000 and 5000 Daltons) of the hydrophilic polymer polyethylene glycol (PEG). Conventional and PEGylated liposomes containing CPT were formulated via the lipid film hydration method (solvent evaporation) using a rotary flash evaporator after optimising various formulation parameters. The following physicochemical characteristics were investigated: surface morphology, particle size, encapsulation efficiency, in vitro release, and formulation stability. Different molecular weights of PEG were used to improve the encapsulation efficiency and particle size. The stealth liposomes prepared with PEG_5000_ were discrete in shape and with a higher encapsulation efficiency (83 ± 0.4%) and a prolonged rate of drug release (32.2% in 9 h) compared with conventional liposomes (64.8 ± 0.8% and 52.4%, respectively) and stealth liposomes containing PEG_2000_ (79.00 ± 0.4% and 45.3%, respectively). Furthermore, the stealth liposomes prepared with PEG_5000_ were highly stable at refrigeration temperature. Significant changes were observed using various pharmacokinetic parameters (mean residence time (MRT), half-life, elimination rate, volume of distribution, clearance, and area under the curve) of stealth liposomes containing PEG_2000_ and PEG_5000_ compared with conventional liposomes. The stealth liposomes prepared with PEG_5000_ showed promising results with a slow rate of release over a long period compared with conventional liposomes and liposomes prepared with PEG_2000_, with altered tissue distribution and pharmacokinetic parameters.

## 1. Introduction

**Figure 1:**
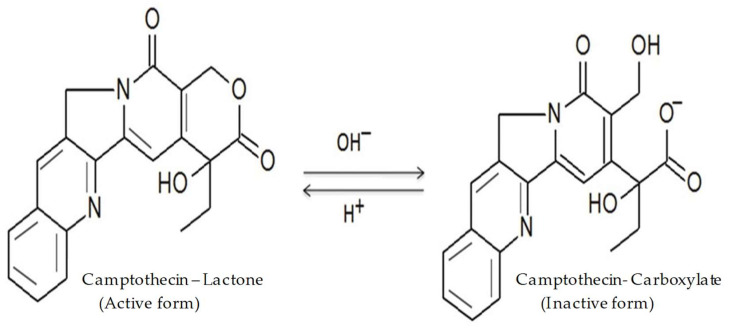
The pH-dependent hydrolysis of Camptothecin.

Camptothecin (CPT) is a naturally occurring quinolone alkaloid found in the bark of *Camptotheca acuminata* and members of the genus *Nothapodytes* [1]. CPT inhibits DNA topoisomerase I via non-covalent interactions, which inhibit the growth of several tumour types. Various studies have revealed that the lactone ring of CPT is essential for its antitumour activity [2]. However, two major drawbacks are related to the efficacy of CPT: its poor solubility in water and the instability of the lactone moiety (Figure 1). Within the physiological environment, the lactone ring is converted to its carboxylate form via a pH-dependent reaction, thereby making the drug less active, highly toxic, and poorly bioavailable [3]. Several approaches have been tested to increase the drug’s solubility and stability. Some of them involve the use of polymeric microspheres, liposomes, micro-emulsions, and the formation of inclusion complexes with cyclodextrin. Thus far, these methods have yielded some success. In particular, liposomes containing a polyethylene glycol (PEG) shell for the injectable delivery of CPT have shown promising results.

Liposomes are spherical, bi-layered lipid vesicles with an encapsulated aqueous phase in the centre. Encapsulation within liposomes increases the in vivo stability, solubility, and bioavailability of poorly soluble drugs. Additionally, encapsulation improves their pharmacokinetics and biodistribution and decreases their toxicity. Liposomes prepared from hydrophilic polymers have gained attention as vehicles for delivering various drugs because of their smaller size, high stability, prolonged release period, and prevention of rapid reticuloendothelial system (RES) clearance [4].

PEG has been widely used as polymeric steric stabiliser among the various polymers investigated. It was reported that liposomes containing a PEG shell reside in the blood circulation for a longer period than non-coated liposomes. This longer circulation time prolongs the exposure of tumour cells to anticancer drugs and improves passive targeting via the enhanced permeation and retention (EPR) effect [5].

PEG possesses high solubility in both aqueous and organic media. Surface modification of liposomes with PEG can be achieved by physically adsorbing the polymer onto the surface of vesicles. PEG possesses a flexible chain that occupies space immediately adjacent to the liposome surface. Cholesterol in liposomal formulations is used as a natural anchor for PEG. The PEG chain is attached to the 3-OH group of cholesterol via an ester or ether linkage. Because of cholesterol’s lipophilicity and structural compatibility with liposomal phospholipids, cholesterol–PEG is easily incorporated into the liposome membranes. While dissolving the lipids and PEG in organic solvents (chloroform, methanol), PEG can covalently bind to the polar head group of phosphatidyl choline (lecithin) [6]. Considering these factors, we chose a PEG segment that generates a hydrophilic shell that stabilises the liposomes through steric repulsion and avoids the use of added surfactants.

The current study focuses on preparing biodegradable PEGylated liposomes to improve the aqueous solubility and stability of CPT. Particles capable of sustained release were produced via the lipid film hydration technique using a rotary flash evaporator with a different drug to polymer ratios. The morphology of CPT-loaded liposomes was studied using scanning electron microscopy (SEM) and transmission electron microscopy (TEM), and the particle size was measured using a Malvern zetasizer. The encapsulation efficiency, in vitro drug release profiles, and in vivo pharmacokinetic study of liposomal formulations were also determined.

## 2. Results and Discussion

CPT was loaded into stealth liposomes via lipid film hydration at various CPT/polymer ratios (1:2.5, 1:5, 1:12.5, 1:25). The CPT-loaded PEG liposomes were characterised by measuring the encapsulation efficiency and in vitro rate of drug release. The PEG chain length was an important parameter to determine the in vitro characteristics of liposomes [7]. Additionally, phosphatidyl choline and cholesterol were used to stabilise the lipid bilayer and improve the structural rigidity of the vesicles by preventing them from fusing and aggregating [8].

The lipid film hydration technique provides certain advantages over similar methods, namely that it is easier to scale up and reduces the chance of drug loss during encapsulation [9,10]. To achieve a uniform particle shape and size as well as to improve the drug loading and encapsulation efficiency, various parameters for the CPT liposomal formulation were optimised in this study. The liposomes were prepared using two PEG formulations with different molecular weights (m.wt 2000 Daltons and 5000 Daltons) to determine whether their length influenced drug loading. Chloroform and methanol were used as the organic co-solvents to dissolve the polymers. Organic solvents were selected based on their miscibility with water, ability to dissolve both CPT and PEG, as well as their low boiling points, which allowed for easy evaporation and the complete removal of solvent [11].

### 2.1. Optimisation of Solvent Evaporation

To evaporate the solvents more quickly and ensure the stability of the formulation, the rotary flash evaporator was attached to a vacuum pump and the temperature was optimised to 40 °C in a water bath to evaporate the solvents at the rotational speed of 100 rpm. The formulations were found to be stable under these conditions. Various rotation speeds (75 rpm, 100 rpm, and 150 rpm) and temperatures (40 °C and 70 °C) were tested. At higher temperatures, the fusion of liposomes occurred and their shapes were not discrete.

### 2.2. Optimisation of Sonication

Sonication was performed using a bath-type sonicator at various times from 2 to 10 min. At 10 min and 5 min, the formulations were found to be non-homogeneous and unstable. Because prolonged sonication may promote the oxidation of lipids, no liposomes were observed at the end of sonication [12]. However, when the sonication time was reduced to 2 min, the liposomes were stable and discrete in shape, showed particle aggregation, and had a smaller particle size. Thus, the sonication time was fixed at 2 min.

### 2.3. Optimisation of Centrifugation

A cooling centrifuge was used to separate the liposomes from free CPT. The preparations were centrifuged at 4032× *g* and 11,200× *g* at 4 °C for 30 min, although the separation of liposomes was not observed. However, after centrifugation at 19,515× *g* and 4 °C for 1 h, the liposomes separated cleanly from the free CPT and were collected and stored for further studies.

For our final optimised liposome preparation, we used two PEG polymers with different molecular weights, a sonication time of 2 min, rotary flask evaporation at 100 rpm with a temperature of 40 °C, and centrifugation at 19,515× *g* for 1 h at 4 °C. These parameters were held constant for all the formulations.

### 2.4. Constructing the CPT Calibration Curve

**Figure 2:**
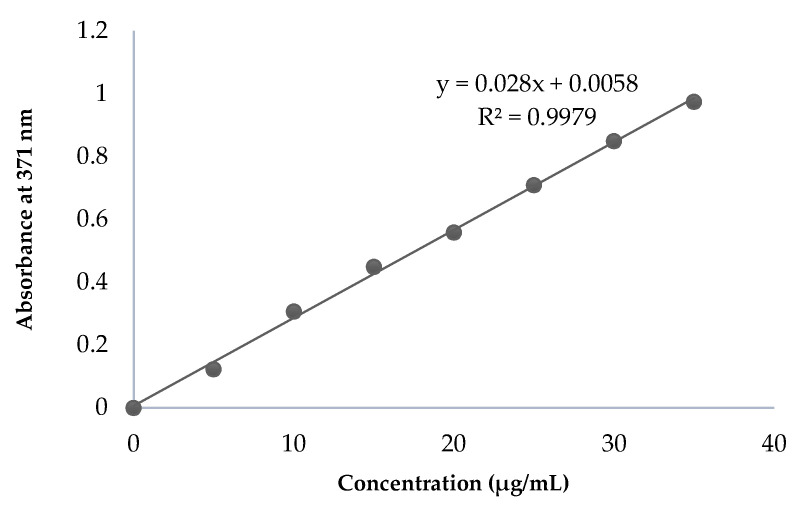
Calibration curve of pure CPT.

The λ_max_ of CPT was found to be 371 nm, which was selected for further analysis. The range of linearity was identified between drug concentrations of 5 and 35 µg/mL CPT (Figure 2).

### 2.5. Morphological Examination and Particle Size Determination

**Figure 3:**
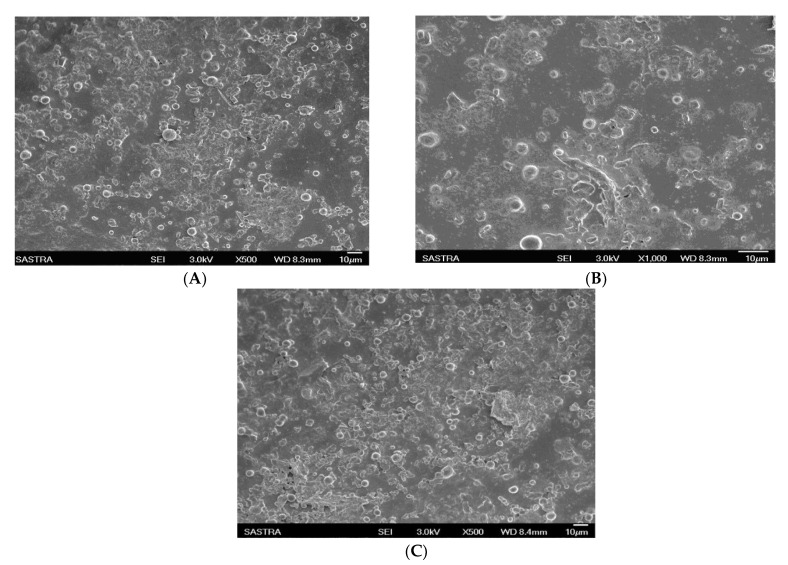
SEM photographs of conventional liposomes and stealth liposomes containing CPT: (**A**) conventional liposomal formulation F4; (**B**,**C**) stealth liposomal formulations F8 and F12, respectively.

A consistent particle size is crucial because it affects the in vivo fate of a particulate drug delivery system. The stealth effect of liposomes is due to their small particle size (<200 nm) and negative zeta potential. The morphology of the liposomes was examined by SEM, as shown in Figure 3A–C, which revealed discrete, round particles. The particle sizes ranged from 0.920 μm to 0.498 μm for formulations F1–F4, 0.332 μm to 0.234 μm for formulations F5–F8, and 0.438 μm to 0.168 μm for formulations F9–F12, as given in Table 1. The CPT liposomes (F12) containing the drug/polymer ratio of 1:25 showed a smaller particle size and narrowed size distribution than the other formulations. The zeta potentials of the liposomes, an important index for the stability of liposomal formulations, ranged from −16.8 ± 2.1 mV to −27.05 ± 0.8 mV (Figure S1). The presence of a negative charge increased the stability and blood circulation of the liposomes.

**Figure 4:**
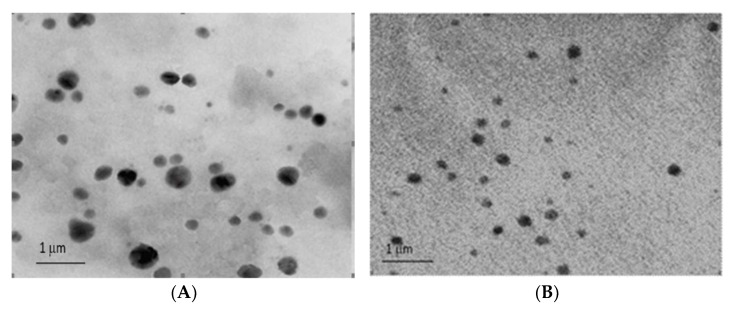
TEM photographs of stealth liposomal formulations of CPT: (**A**) formulation F8; (**B**) formulation F12.

Furthermore, the TEM images (Figure 4A,B) showed that the stealth liposomes were spherical with a uniform distribution and smooth outer morphology.

### 2.6. Entrapment Efficiency

The factors that affect the encapsulation efficiency of liposomes prepared via lipid film hydration are as follows:

(a) The affinity between the drug and polymer;
(b) The volume of the hydrophobic core;
(c) The ability of the drug to self-aggregate [13].

Among these three, the affinity between the drug and core-forming block is considered the main factor. To obtain liposomes with a long blood circulation time, 12 formulations containing different ratios of drug and polymer were prepared. The encapsulation efficiency values for CPT were 79 ± 0.4% for liposomes containing a 1:25 ratio (drug to polymer) of PEG_2000_ (F8) and 83.0 ± 0.4% for formulations containing a 1:25 ratio of PEG_5000_ (F12), as given in Table 1. In our study, liposomes prepared with a 1:25 ratio of polymer showed a higher encapsulation efficiency than those prepared with 1:5 and 1:12.5 ratios of the same polymer. Drug/polymer ratios higher than 1:25 caused particle aggregation and a very low liposomal yield [14]. This finding is important because the nature of the drug loaded into the liposomes may determine the rate of release from the formulations. Hence, the formulations showing a lower encapsulation efficiency were excluded from further studies.

### 2.7. In Vitro Release Studies

The in vitro characteristics of a liposome-encapsulated lipophilic drug largely depend on the lipophilic properties of the core shell. The other factors influencing the rate of drug release include the following [14]:

(a) Type of release medium used;
(b) Physicochemical properties of the polymer;
(c) Internal structure of the liposome.

In vitro studies were performed for conventional and stealth liposomal formulations with the highest encapsulation efficiencies (F4, F8, and F12) and compared with the standard CPT solution. In this test, PBS was used as the release medium to investigate other factors affecting the release profile. All liposomal formulations exhibited an initial burst release of CPT from the dialysis bag, thereby showing that the release lag time induced by the dialysis bag could essentially be neglected [15]. The cumulative percentage of CPT solution released within 6 h was found to be 96.6 ± 0.6% at 37 °C in PBS. For the liposomal formulations containing CPT, the percentages of released CPT were 45.63 ± 0.9%, 32.07 ± 0.9%, and 25.39 ± 0.1% at 6 h for F4, F8, and F12, respectively.

**Figure 5:**
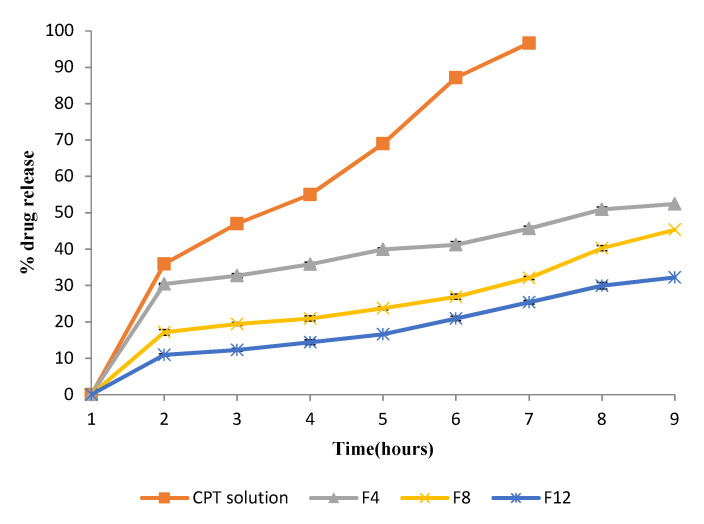
In vitro drug release from CPT liposomal formulations (F4, F8, F12). Values are expressed as means ± SD.

At 8 h, the percentages of the released drug increased to 52.41 ± 0.8% (F4), 45.32 ± 0.6% (F8), and 32.22 ± 0.4% (F12), as shown in Figure 5. These results showed that the introduction of PEG polymers to liposomes led to a decreased release rate compared with conventional liposomes (F4). Additionally, formulation F12 showed a slower rate of release than formulation F8. The results indicate that the stealth liposomes of CPT exhibited slow release when compared to the conventional liposomes, due to the adsorption of hydrophilic PEG and the rigidity of the lipid bilayer [16]. All the liposomal formulations prepared using the lipid hydration technique had a uniform size without aggregation and showed slow release over a prolonged period [17]. The slow, steady release of CPT observed with these preparations demonstrates that our method promotes efficient encapsulation.

### 2.8. Drug Release Kinetics

To study the release kinetics of CPT, data obtained from the in vitro release studies were plotted using various kinetic models, including the zero-order, first-order, Higuchi, and Korsmeyer–Peppas models [18]. The in vitro release of CPT from liposomes was best explained by Higuchi’s equation, as this model showed the highest linearity (highest r^2^ and lowest Akaike information criterion (AIC)), followed by the first-order model for all liposomal formulations.

The release of the drug was proportional to the square root of time, demonstrating that its release was diffusion-controlled [19]. The obtained data were also plotted using the Korsmeyer–Peppas model to calculate the n value, which showed that the release of the drug from all formulations was diffusion-controlled, as shown in Table 2.

### 2.9. Standard Plot of Camptothecin in Spiked Plasma

**Figure 6:**
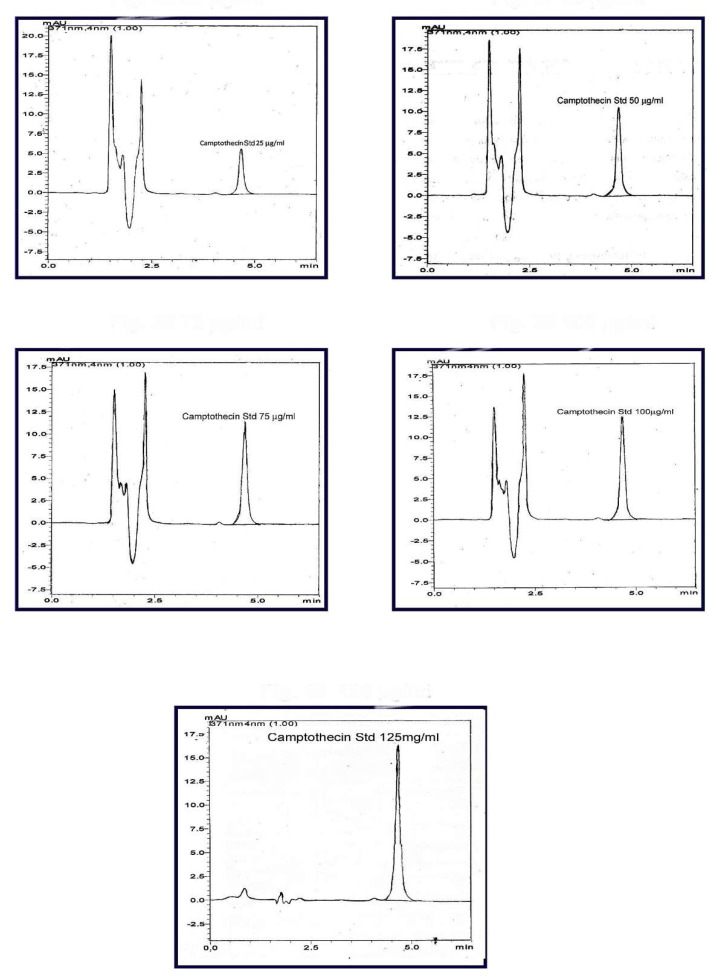
HPLC chromatogram of camptothecin in spiked plasma (concentration range of 25–125 μg/mL).

**Figure 7:**
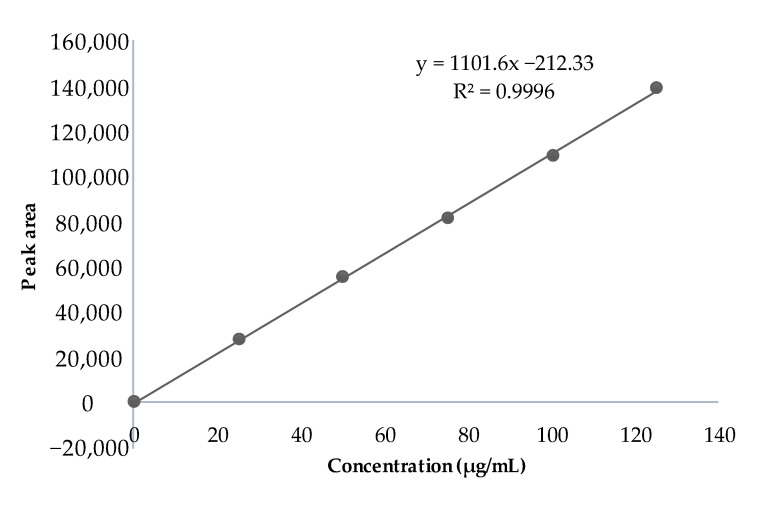
Standard plot of CPT in spiked plasma obtained via HPLC method.

The peak area was noted from the chromatogram and plotted against the concentration to obtain the standard curve. The HPLC chromatogram of CPT in spiked plasma is shown in Figure 6. Linearity was obtained in the concentration range of 25–125 μg/mL, as given in Figure 7.

### 2.10. In Vivo Pharmacokinetic Study

**Figure 8:**
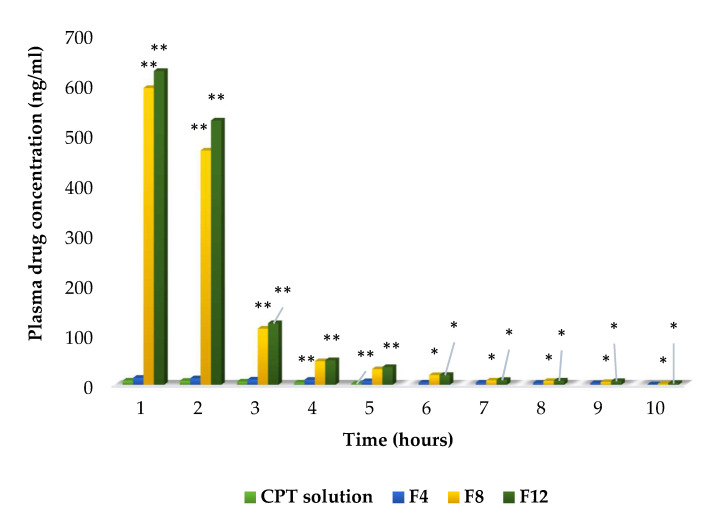
Plasma drug concentrations of CPT drug solution, conventional (F4), and PEGylated (F8, F12) liposomes (in vivo pharmacokinetic study). Note: ** denotes *p* < 0.01, * denotes *p* < 0.05 when compared with CPT solution (ANOVA followed by post hoc Dunnett’s test).

The liposomal formulations F4, F8, and F12 were chosen for the in vivo pharmacokinetic study due to their smaller particle size, low polydispersity index, higher entrapment efficiency, and slower rate of release. The PEGylated liposomes initially showed higher circulating levels in the blood than the free CPT solution. At 5 h, the concentrations of F4, F8, and F12 in the blood were approximately 7.2 ng/mL, 31 ng/mL, and 35.4 ng/mL, respectively. In contrast, the free CPT had been removed from the circulation and could not be detected at 5 h (Figure 8). Formulations F4, F8, and F12 remained in the blood circulation for 10 h and showed delayed blood clearance. The formulations F8 and F12 extended the half-life of CPT to 4.3 h and 5.9 h, respectively. The area under the curve (AUC) was high in PEGylated liposomes; specifically, formulation F12 showed the highest AUC, indicating a significant increase in bioavailability exhibited by this formulation compared with free CPT and formulations F4 and F8. The pharmacokinetic parameters are shown in Table 3.

As PEG_5000_ (F12, 1:25) liposomes had longer PEG chains and a more rigid structure, they showed the highest drug-loading efficiency. Furthermore, F12 had a slow rate of release and a longer half-life than the other conventional and PEGylated formulations [20]. Significant increases (*p* < 0.01) in the AUC and mean residence time (MRT) were observed in F12 compared with those in CPT solution and conventional liposomes. Additionally, the clearance (7.57 mL/min/kg) and volume of distribution (690.5 mL/kg) were found to be significantly lower (*p* < 0.01) than those obtained with other formulations. The increased MRT in plasma compared with the same dose of CPT solution and conventional liposomes could be due to the coating of PEG on the surfaces of liposomes and the sustained release of CPT from PEGylated formulations [21,22]. PEGylation with high-molecular-weight PEG decreased the volume of distribution (V_d_), slowed the elimination of PEGylated liposomal CPT, and increased the area under the curve [23].

The in vivo characteristics of PEGylated liposomes were consistent with their in vitro physicochemical characteristics. This study confirmed that PEGylated liposomes enhance the drug stability and longevity of CPT in the blood by preventing the adsorption of various blood components onto their surfaces.

### 2.11. Tissue Distribution Studies

**Figure 9:**
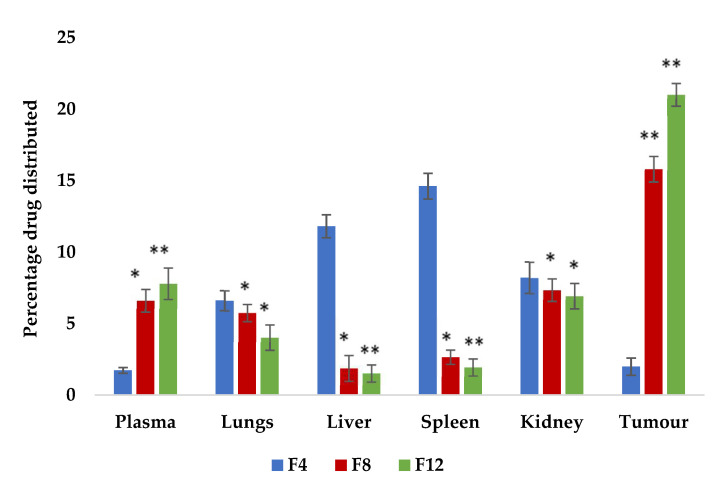
CPT tissue distribution in mice bearing EAC cells 24 h after i.v. injection of CPT-loaded PEGylated liposomes (F8 and F12) at a dose of 2.5 mg/kg. Each value represents the mean ± SD (*n* = 3), ** *p* < 0.01, * *p* < 0.05 compared with conventional liposome formulation (F4) (one-way ANOVA followed by post hoc Dunnett’s test).

The tissue distribution profiles of CPT-loaded conventional and stealth liposomes after i.v. injection of 2.5 mg/kg into mice bearing EAC are shown in Figure 9. A tissue distribution study was conducted to gain a deeper insight into the in vivo behavior of the prepared CPT liposomes and to better elucidate the reasons why drug-loaded PEGylated liposomes had superior antitumour efficacy than conventional CPT liposomes. Considering that the tissue distribution in tumour-bearing animals may be different from normal animals due to certain physiological changes brought about by tumour development, EAC-bearing mice were employed instead of normal mice in the tissue distribution investigations.

The rapid accumulation of liposomes in the various organs was observed for all formulations. The blood plasma levels of CPT-loaded liposomes containing F8 and F12 were relatively higher (6.6% and 7.80%, respectively) than formulation F4 (1.72%). Liver samples showed significantly decreased uptake of CPT liposomes containing F8 and F12 (1.88% and 1.52%, respectively) when compared to F4 (11.8%). The tumour accumulation rates of CPT-loaded liposomes of F8 and F12 were higher (15.8% and 21%, respectively) than for F4 (1.98%). These results showed that the PEGylated liposomes possess the ability to deliver large amounts of CPT, in its most active form, to the tumour site via passive targeting with a long-circulating carrier [24].

The CPT concentrations in the lungs (5.75% and 4.02% for F8 and F12, respectively) were also high, possibly due to the filtration effect of the lung capillary bed removing some large particles or their aggregates. Regarding the concentrations in the kidneys, one of the major elimination pathways of the original CPT was found to be low and showed a significant difference between the groups treated with CPT-loaded liposomes F8 and F12 when compared with conventional liposome formulation F4. However, the CPT concentrations in the liver, a major RES organ, were significantly (*p* < 0.01) lower (1.88% and 1.52% for F8 and F12, respectively), which might be attributed to the steric stabilisation provided by the PEG shell and could be viewed as proof of the RES-evading ability of PEGylated liposomes. Liposomes surface-modified with PEG can stay in circulation much longer, resulting in considerably higher blood concentrations of the loaded drug for prolonged periods and a passive targeting effect. When compared with conventional camptothecin liposomes (F4), formulations F8 and F12 showed highly significant results due to PEGylation.

Therefore, PEGylated liposomes with smaller sizes seem to be more capable of staying in circulation for longer periods, which might be advantageous in the delivery of antitumour drugs. The results obtained here indicate that by utilising the PEGylated liposomes as drug carriers, the blood concentration of CPT could be maintained for a long period and the tissue distribution could also be altered. An optimised tissue distribution may lead to improved drug efficacy and reduced side effects.

### 2.12. Physical Stability Analysis

The stability study results indicated that stealth liposomal formulations (F8 and F12) were highly stable when stored at refrigeration temperature (2–8 °C). Significant changes in particle size, PDI, and zeta potential were observed after 30 days for the conventional liposomes stored at room temperature and at 2–8 °C, whereas the PEGylated liposomes were more stable after 30 days of preservation at 2–8 °C than the conventional liposomes, as shown in Table 4. Zeta potential values of PEGylated liposome samples were found to be stable or slightly reduced after their preservation at 2–8 °C, showing that their tendency for aggregation was greatly decreased.

After 30 days, the encapsulation efficiencies for F8 and F12 were 76.69 ± 0.9% and 80.19 ± 0.6%, respectively, at refrigeration temperature. In contrast, the conventional formulations had an encapsulation efficiency of 60.14 ± 0.4% after 30 days. Furthermore, all of the formulations were found to be unstable at room temperature after 30 days, with entrapment efficiencies of 48.31 ± 0.3%, 69.11 ± 0.6%, and 69.68 ± 0.5% for liposomal formulations F4, F8, and F12, respectively, as shown in Table 5. The lowered entrapment efficiency observed at room temperature may have been due to drug expulsion from the liposomes. Additionally, the formulations showed no change in colour during the study period.

## 3. Materials and Methods

### 3.1. Materials

The following materials were used in the study: camptothecin (CPT), S-(+)-CPT, approximately 95% by HPLC (Sigma Chemical Co., St. Louis, MO, USA), egg L-alpha-phosphatidylcholine ≥99% (L-alpha-lecithin), cholesterol ≥99% (Avanti Polar Lipids Inc., Alabaster, AL, USA), PEG (M.W. 2000 and 5000 Daltons; Sigma Chemical Co., St. Louis, MO, USA), chloroform and methanol (Merck and Co., Kenilworth, NJ, USA), phosphate-buffered saline (pH 7.4; Sigma Chemicals, St. Louis, MO, USA), dialysis tubing membrane (M.W. cut off 12,000–14,000 Daltons), and dialysis tubing closures (Sigma Aldrich, St. Louis, MO, USA). The chemicals used were of analytical grade.

### 3.2. Methods

#### 3.2.1. Preparation of CPT-Loaded Conventional and PEGylated Liposomes

Liposomes were developed via the lipid film hydration technique with a rotary flash evaporator [25]. Various concentrations of the phospholipids, cholesterol, and PEG were dispersed in 10 mL of a solvent mixture of chloroform and methanol along with 25 mg of CPT. Next, the solvents (chloroform: methanol 3:2 *v/v*) were evaporated using a rotary flash evaporator at 40 °C under reduced pressure (Rotavapor R-215, Buchi, vacuum controller V-855) for 1 h to form a thin lipid layer. The film deposited on the round bottom flask wall was rehydrated by adding 10 mL of phosphate-buffered saline solution at pH 7.4 under continuous agitation to form a suspension of multilamellar liposomes. The suspension was then sonicated using a bath-type sonicator (5 s sonication followed by 1 s standby) (Ultrasonic set, WiseClean WUC-D10H, Korea; frequency: 40 kHz; amplitude: 100%) to form unilamellar vesicles [26]. The same method was adopted to prepare conventional liposomes without PEG (Table 6).

#### 3.2.2. Constructing the CPT Calibration Curve

Spectrophotometry was used to create a calibration curve to determine the concentration of encapsulated CPT. The CPT solution was prepared by dissolving 25 mg of drug in 25 mL of PBS at pH 7.4 to create a 1 mg/mL solution. From the above solution, 10 mL was removed and diluted to 100 mL with PBS to a final concentration of 100 µg/mL. The above working stock solution was diluted serially to obtain the CPT solutions with the following concentrations: 5, 10, 15, 20, 25, 30, and 35 µg/mL. The absorbance of each diluted solution was measured at 371 nm using a Shimadzu UV–visible spectrophotometer. PBS was used as a blank. The absorbance values were plotted against the CPT concentrations.

#### 3.2.3. Determination of the Particle Size, Polydispersity Index, Zeta Potential, and Surface Morphology

A zetasizer (Nano-ZS, Malvern Instruments, Malvern, UK) was used to determine the size and polydispersity index of liposome particles. The zeta potential was determined by appropriately diluting the formulations in a clear disposable polycarbonate capillary cell. The morphological characteristics of the liposomal vesicles were determined using SEM (Jeol-JF1600 field emission SEM). The morphology and particle size of stealth liposomes were further confirmed by TEM (Jeol, 1200Ex II, Tokyo, Japan).

#### 3.2.4. Determination of Entrapment Efficiency

The CPT-loaded liposomes were centrifuged at 13,200 rpm at 4 °C for 1 h (Cooling centrifuge; model 3K; Sigma, Osterode am Harz, Germany) to separate the drug-loaded liposomes from the unencapsulated drug. The supernatant was collected and diluted appropriately. The quantity of CPT loaded into liposomes was determined by measuring the absorbance at 371 nm and using the aforementioned calibration curve. The encapsulation efficiency was calculated using the following formula [27]:

$$\;\%\mathrm{EE}=\frac{\mathrm{Total}\;\mathrm{amount}\;\mathrm{of}\;\mathrm{drug}-\mathrm{Drug}\;\mathrm{in}\;\mathrm{supernatant}}{\mathrm{Total}\;\mathrm{amount}\;\mathrm{of}\;\mathrm{drug}}\;\times \;100$$

#### 3.2.5. In Vitro Release of CPT from Conventional and Stealth Liposomes

The drug release of CPT from liposomes was determined using a diffusion technique involving a dialysis bag. The formulation (5 mL) was introduced into a dialysis tubing membrane bag (M.W. cut-off of 12,000–14,000 Daltons) and sealed with closure clips. The bag was immersed in 100 mL of PBS at pH 7.4, and the temperature was maintained at 37 ± 2 °C with gentle stirring. At fixed time intervals, the samples were collected from the release medium, replaced with fresh buffer, and analysed by measuring the absorbance at 371 nm using the UV spectrophotometer. The samples were compared with a CPT standard solution (the CPT solution was prepared by dissolving the pure drug in a 10 mL mixture of polyethylene glycol 400, propylene glycol, and Tween 80 (40:58:2)) [28]. and analysed under the same experimental conditions, then the percentage of released drug was calculated. All formulations were analysed in triplicate [29].

#### 3.2.6. In Vitro Release Kinetics of CPT from Liposomes

The data obtained from in vitro drug release studies were plotted using various kinetic models—namely zero-order (cumulative amount of drug released vs. time), first-order (log cumulative percentage of drug remaining vs. time), and Higuchi models (cumulative percentage of drug released vs. the square root of time)—to study the kinetics of drug release from each formulation [30].

To evaluate the mechanism of drug release, the obtained data were plotted using the Korsmeyer equation (log cumulative percentage of drug released vs. log time), and the exponent n was calculated from the straight line and Hixson–Crowell model (cube root percentage of the drug remaining vs. time) [31].

#### 3.2.7. Standard Plot of Camptothecin in Spiked Plasma Assessed by HPLC

The CPT stock solution containing 1000–5000 μL of drug was prepared. To each of these solutions, 100 μL was spiked into 100 μL of each plasma sample in an Eppendorf tube to obtain different concentrations. These solutions were then precipitated with 200 μL of methanol. The obtained solution was centrifuged in a cooling centrifuge at 10,000 rpm and the supernatant was injected into the HPLC system. The samples were eluted at room temperature with a mobile phase consisting of 60:40 mixtures of triethylamine-acetate buffer (pH 5) and acetonitrile. The flow rate was set at 1 mL/min and the CPT was monitored at 371 nm. The peak area was noted and plotted against the concentration used to obtain the standard curve.

#### 3.2.8. In Vivo Pharmacokinetic Characteristics of CPT Liposomes

The study was approved by the Institutional Animal Ethical Committee (Registration number 158/1999/CPCSEA). Healthy male rabbits (1.5–2 kg body weight) were used for pharmacokinetic investigations. The animals were housed in temperature-controlled rooms on a 12-h light/dark cycle. The animals were given access to food and water ad libitum. Four treatment groups were used to determine the in vivo pharmacokinetic parameters: group 1 was treated with CPT solution, group 2 was treated with F4 (conventional liposomes), group 3 was treated with F8 (PEG_2000_ stealth liposomes), and group 4 was treated with F12 (PEG_5000_ stealth liposomes). Conventional and stealth liposomes were dispersed in a 0.9% sodium chloride solution at the required concentration before administration. Solutions of CPT with the same concentrations were prepared simultaneously in identical vehicles by diluting the CPT solution. All formulations were sterile-filtered before injection into the rabbits. The animals were injected intravenously with a dose of 2.5 mg of CPT/kg body weight. After administration, blood samples were collected at various time intervals from the ear vein and centrifuged at 13,500 rpm for 4 min to separate the plasma (100 μL), then CPT was extracted using a chloroform/methanol solvent mixture (4:1 *v/v*). After extraction, 25 μL of the chloroform/methanol layer was directly injected into the high-performance liquid chromatography (HPLC) system (Shimadzu LC-20AT) to determine the concentration of CPT. The separation was performed using a Luna 5 μm phenyl-hexyl column (Phenomenex, USA) with UV detection at 352 nm [32]. All analyses were carried out in triplicate. The mobile phase was 40:60 (*v/v*) acetonitrile and ammonium acetate buffer with 2% triethylamine (pH adjusted to 5 with 5% glacial acetic acid). The flow rate was set to 1 mL/min. The concentration of drug was calculated from the standard plot.

Various pharmacokinetic parameters, such as the area under the curve (AUC), volume of distribution (V_d_), clearance (Cl), half-life (t_1/2_), mean residence time (MRT), and elimination rate constant (K_el_), were calculated based on a non-compartment model (WinNonlin 5.2.1 version).

#### 3.2.9. Tissue Distribution Studies of CPT Liposomes in Ehrlich Ascites Carcinoma (EAC)-Bearing Mice

Tissue distribution studies of PEGylated liposomes containing CPT were compared with conventional CPT liposomes in mice bearing Ehrlich ascites carcinoma. Healthy male mice with body weights between 20 and 22 g were used for body distribution investigations. Animals were given adequate amounts of food and water and were housed in temperature controlled rooms. Animals were divided into 3 groups, each consisting of 3 animals. The mice in group 1 being treated with CPT liposomal formulation F4, those in group 2 were treated with formulation F8, and those in group 3 were treated group with camptothecin liposomes containing formulation F12.

Based on various literature studies, the tolerability of the CPT dose and duration of treatment were established prior to initiating the in vivo studies. CPT formulations (F4, F8, and F12) were administered to the tumour-bearing mice by single bolus i.v. injection in to the lateral tail vein at a dose of 2.5 mg/kg. After 24 h of injection, animals were sacrificed. Blood samples (0.5 mL) were collected from each animal by cardiac puncture and plasma samples were collected in new tubes. Different organs such as the heart, liver, spleen, lungs, kidneys and tumour were removed, weighed, and homogenised. Both the plasma samples and tissue homogenates were stored at 4 °C for 12 h [32].

Then, the CPT concentration was determined using HPLC. Here, 300 µL ice-cold methanol was added to 100 µL plasma or other tissue-homogenised solution to precipitate plasma proteins and solubilise the liposomes. The samples were centrifuged at 10,000 rpm for 30 min at 10 °C. The methanolic solution was stored at 4 °C until analysis. The clear supernatant was injected into a Phenomenex Luna 5 µ phenyl-hexyl column and eluted with triethylamine-acetate buffer/acetonitrile (40:60) as a mobile phase at a flow rate of 1 mL/min, then the peak area was determined at 371 nm and the concentration of CPT was quantified.

#### 3.2.10. Physical Stability Analysis

The liposomal formulations were sealed in 20 mL glass vials and exposed to various temperatures, including refrigeration temperature (2–8 °C) and normal room temperature (28 ± 2 °C), for a short period of four weeks. Samples were collected, appropriate dilutions were made using PBS pH 7.4, and the samples were analysed by measuring the absorbance at 371 nm using a UV spectrophotometer. The encapsulation efficiency was calculated using the previously mentioned equation to determine the leakage of drug from the formulations. Colour changes in the formulations were analysed visually by keeping the samples in a dark background.

#### 3.2.11. Statistical Analysis

Values were expressed as the means ± standard deviation (SD) and were statistically analysed using one-way analysis of variance (ANOVA), followed by post hoc Dunnett’s test. Values of *p* < 0.05 were deemed to be statistically significant.

## 4. Conclusions

Numerous attempts to overcome the poor water solubility of CPT by various nano drug delivery systems are described in various sources in the literature. However, the results of these approaches may be hampered by incomplete separation of free CPT from the formulations. Since this issue has not been investigated in detail, we developed CPT-containing liposomes from the PEG polymers at two molecular weights. We measured their encapsulation efficiency, analysed their in vitro release behaviour, and compared the in vivo pharmacokinetic properties and tissue distribution with conventional liposomes. The current study has proven that the composition of PEG polymers with high molecular weight and various formulation parameters influenced the particle size and encapsulation efficiency. The small particle size and higher encapsulation efficiency helped sustain the CPT release and prolonged the in vivo blood circulation time with altered tissue distribution. Our study revealed that PEGylated liposomes could be used as promising delivery systems to prolong the release and improve the stability of the lipophilic anticancer drug CPT. Further, the addition of PEGs and lipids to the formulation needs to be investigated, in terms of its influence on the incorporation capacity and retention ability. The stealth liposomes represent a realistic approach to the selective drug delivery of CPT in various type of cancers and opens up new avenues for therapeutic applications. Understanding the advances in liposomal technology and the challenges that still need to be controlled will allow future research of liposomes to improve on existing platforms.

## Figures and Tables

**Table 1:** Particle size, polydispersity index (PDI), zeta potential, and entrapment efficiency of the liposomes containing CPT.

Formulation Code	* Mean Particle Diameter (µm)	* PDI	Zeta Potential (mV)	* Entrapment Efficiency (%)
F1	0.920 ± 0.10	0.31 ± 0.02	−17.8 ± 1.9	50.30 ± 0.6
F2	0.856 ± 0.09	0.34 ± 0.04	−19.25 ± 2.6	59.02 ± 0.5
F3	0.745 ± 0.17	0.41 ± 0.09	−16.8 ± 2.1	62.75 ± 0.5
F4	0.498 ± 0.16	0.29 ± 0.03	−20.2 ± 0.9	64.80 ± 0.8
F5	0.332 ± 0.05	0.44 ± 0.08	−17.25 ± 3.0	60.21 ± 0.8
F6	0.296 ± 0.11	0.46 ± 0.06	−19.5 ± 2.2	70.89 ± 0.7
F7	0.241 ± 0.13	0.52 ± 0.07	−20.55 ± 1.6	75.50 ± 0.5
F8	0.234 ± 0.19	0.47 ± 0.06	−23.86 ± 1.8	79.00 ± 0.4
F9	0.438 ± 0.15	0.46 ± 0.11	−22.84 ± 1.1	74.04 ± 0.5
F10	0.407 ± 0.21	0.45 ± 0.03	−25.6 ± 1.4	77.12 ± 0.3
F11	0.369 ± 0.23	0.51 ± 0.12	−24.9 ± 1.2	79.52 ± 0.3
F12	0.168 ± 0.09	0.28 ± 0.09	−27.05 ± 0.8	83.00 ± 0.4

* Each value was an average of three determinations. Values are expressed as means ± SD.

**Table 2:** Determination of release kinetics of CPT from liposomes.

Formulation Code	Higuchi		Korsmeyer- Peppas			Zero Order			First Order			Hixson-Crowell		
	r^2^	AIC	r^2^	*n* Value	AIC	r^2^	k_0_ (h^−1^)	AIC	r^2^	K_1_ (h^−1^)	AIC	r^2^	*n* Value	AIC
F4	0.994	35.14	0.998	0.312	40.95	0.984	4.98	47.56	0.991	0.122	42.74	0.996	0.133	51.89
F8	0.990	35.36	0.996	0.288	38.45	0.961	6.88	47.13	0.997	0.098	42.59	0.998	0.145	50.36
F12	0.993	34.35	0.994	0.291	38.23	0.984	7.45	45.92	0.989	0.112	40.25	0.996	0.125	49.75

Note: r^2^ denotes coefficient of determination; AIC denotes Akaike information criterion; k_0_ denotes zero order release constant; K_1_ denotes first order release constant; n denotes diffusion release exponent.

**Table 3:** Pharmacokinetic parameters of CPT-containing liposomes.

Parameters	Drug in Solution	F4 (Conventional)	F8 (PEG_2000_, 1:25) (PEGylated)	F12 (PEG_5000_, 1:25) (PEGylated)
AUC _0-last_ (ng/mL.h)	34.6	64.17 *	3319.3 **	3612.2 **
t_1/2_ (h)	0.81	3.4 *	4.3 *	5.9 **
MRT(h)	0.9	3.79 *	4.92 *	6.1 **
Total clearance (ml/min/kg)	61.2	39.3 *	9.23 **	7.57 **
V_d_ (mL/kg)	1975.2	1833.2 *	1002.5 **	690.5 **
K_el_ (min^−1^)	0.95	0.43 *	0.21 **	0.19 **

Pharmacokinetic parameters of CPT liposomes and CPT solution following i.v. administration in rabbits. Note: ** denotes *p* < 0.01, * denotes *p* < 0.05 when compared with CPT solution (ANOVA followed by post hoc Dunnett’s test).

**Table 4:** Physical stability analysis (particle size, PDI, and zeta potential) of CPT-containing liposomes.

Formulation Code	Mean Particle Diameter (µm)	Mean Particle Diameter After 30 Days (µm)		Poly Dispersity Index (PDI)	PDI after 30 Days		Zeta Potential (mV)	Zeta Potential after 30 Days (mV)	
	Initial	RT	2–8 °C	Initial	RT	2–8 °C	Initial	RT	2–8 °C
F4	0.498 ± 0.16	0.71 ± 0.1	0.62 ± 0.11	0.29 ± 0.03	0.54 ± 0.01	0.40 ± 0.01	−20.2 ± 0.9	−17.9 ± 1.9	−18.8 ± 0.8
F8	0.234 ± 0.19	0.45 ± 0.02	0.24 ± 0.1	0.47 ± 0.06	0.58 ± 0.08	0.49 ± 0.05	−23.86 ± 1.8	−22.11 ± 2.1	−22.85 ± 1.3
F12	0.168 ± 0.09	0.29 ± 0.05	0.19 ± 0.09	0.28 ± 0.09	0.39 ± 0.08	0.28 ± 0.08	−27.05 ± 0.8	−25.18 ± 0.8	−27.01 ± 0.6

Data are given as mean ± SD (*n* = 3).

**Table 5:** Physical stability analysis (entrapment efficiency) of CPT-containing liposomes.

Formulation Code	% Entrapment Efficiency	% Entrapped Drug after 15 Days		% Entrapped Drug after 30 Days	
	Initial	RT	2–8 ° C	RT	2–8 °C
F4	64.8 ± 0.8	55.91 ± 0.6	61.51 ± 0.5	48.31 ± 0.3	60.14 ± 0.4
F8	79 ± 0.4	74.01 ± 0.9	78.01 ± 0.4	69.11 ± 0.6	76.69 ± 0.9
F12	83 ± 0.4	78.56 ± 0.7	80.59 ± 0.5	69.68 ± 0.5	80.19 ± 0.6

Data are given as means ± SD (*n* = 3).

**Table 6:** Formulation of conventional and stealth liposomes containing camptothecin (CPT).

Drug/Polymer	Formulation Code	Drug: Polymer (PEG) Ratio	Amount of Cholesterol (mg)	Amount of Lecithin (mg)	Organic Solvents Used
CPT (Conventional liposomes)	F1	1:0	20	280	Chloroform/methanol (3:2 *v/v*), 10 mL
	F2	1:0	80	500	
	F3	1:0	40	100	
	F4	1:0	50	390	
CPT- PEG_2000_ (Stealth liposomes)	F5	1:2.5	20	280	Chloroform/methanol (3:2 *v/v*), 10 mL
	F6	1:5	80	500	
	F7	1:12.5	40	100	
	F8	1:25	50	390	
CPT- PEG_5000_ (Stealth liposomes)	F9	1:2.5	20	280	Chloroform/methanol (3:2 *v/v*), 10 mL
	F10	1:5	80	500	
	F11	1:12.5	40	100	
	F12	1:25	50	390	

## Data Availability

Not applicable.

## Supplementary Materials

The following supporting information is available online, Figure S1. Zeta sizer report of stealth liposomes containing CPT. (A), (B) represents the stealth liposomal formulations F8 and F12 respectively.

## Sample Availability

Samples are not available from the authors.
