# Supplementary material for: Stealth Liposomes (PEGylated) Containing an Anticancer Drug Camptothecin: In Vitro Characterization and In Vivo Pharmacokinetic and Tissue Distribution Study
Source: Molecules. 2022 Feb 6;27(3):1086. doi: 10.3390/molecules27031086 (PMC8838228; doi:10.3390/molecules27031086)
Supplement: Supplementary file 1 [file molecules-27-01086-s001.zip › molecules-1567705-supplementary.pdf]

SUPPLEMENTARY MATERIAL – ZETA SIZER REPORT

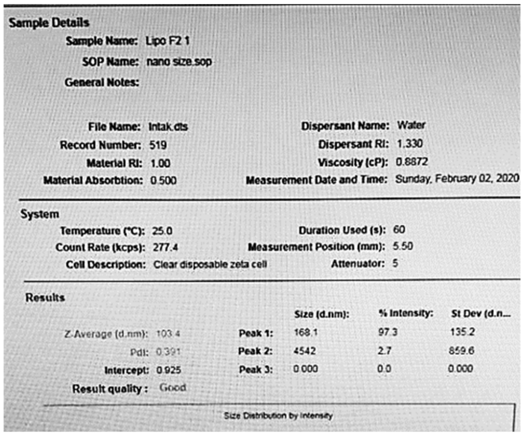

Sample Details

Sample Name: Lipo F2 1
SOP Name: nano size.sop
General Notes:

File Name: Intak.dts
Record Number: 519
Material RI: 1.00
Material Absorbtion: 0.500
Dispersant Name: Water
Dispersant RI: 1.330
Viscosity (cP): 0.8872
Measurement Date and Time: Sunday, February 02, 2020

System

Temperature (°C): 25.0
Count Rate (kcps): 277.4
Cell Description: Clear disposable zeta cell
Duration Used (s): 60
Measurement Position (mm): 5.50
Attenuator: 5

Results

			Size (d.nm):	% Intensity:	St Dev (d.n...
Z-Average (d.nm):	103.4	Peak 1:	168.1	97.3	135.2
PdI:	0.391	Peak 2:	4542	2.7	859.6
Intercept:	0.925	Peak 3:	0.000	0.0	0.000
Result quality :	Good				

Size Distribution by Intensity

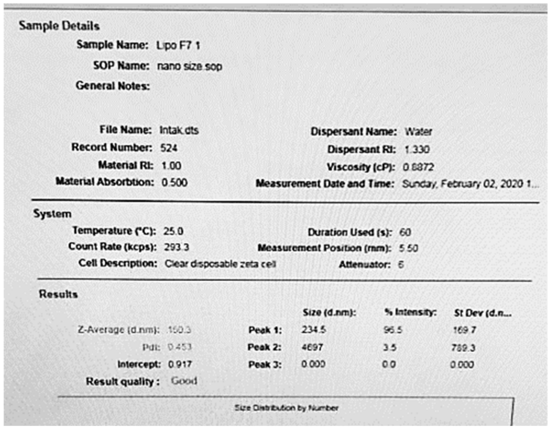

Sample Details

Sample Name: Lipo F7 1
SOP Name: nano size.sop
General Notes:

File Name: Intak.dts
Record Number: 524
Material RI: 1.00
Material Absorbtion: 0.500
Dispersant Name: Water
Dispersant RI: 1.330
Viscosity (cP): 0.8872
Measurement Date and Time: Sunday, February 02, 2020 1...

System

Temperature (°C): 25.0
Count Rate (kcps): 293.3
Cell Description: Clear disposable zeta cell
Duration Used (s): 60
Measurement Position (mm): 5.50
Attenuator: 6

Results

			Size (d.nm):	% Intensity:	St Dev (d.n...
Z-Average (d.nm):	150.3	Peak 1:	234.5	96.5	169.7
PdI:	0.453	Peak 2:	4697	3.5	789.3
Intercept:	0.917	Peak 3:	0.000	0.0	0.000
Result quality :	Good				

Size Distribution by Number

(A) **(B)**

Zeta sizer report of stealth liposomes containing CPT.

(A), (B) represents the stealth liposomal formulations F8 and F12 respectively.
